# Monocyte-to-lymphocyte ratio as a predictor of left ventricular aneurysm in acute STEMI patients

**DOI:** 10.3389/fendo.2025.1701964

**Published:** 2026-03-02

**Authors:** Dong Hu, Dongyang Wu, Ting Huang, Guangji Wang, Xin Guo, Qinshuo Zhao, Man-Hua Chen, Yi Zhou

**Affiliations:** 1 Department of Cardiology, The Central Hospital of Wuhan, Tongji Medical College, Huazhong University of Science and Technology, Wuhan, China; 2 Key Laboratory for Molecular Diagnosis of Hubei Province, The Central Hospital of Wuhan, Tongji Medical College, Huazhong University of Science and Technology, Wuhan, China; 3 Hubei Provincial Engineering Research Center of Intestinal Microecological Diagnosis and Treatment Technology and Clinical Application, Wuhan, Hubei, China; 4 Department of Cardiology, Renmin Hospital of Wuhan University, Wuhan, China

**Keywords:** acute ST-segment elevation myocardial infarction, left ventricular aneurysm, monocyte-to-lymphocyte ratio, predictor, risk

## Abstract

**Background:**

The monocyte-to-lymphocyte ratio (MLR) has emerged as a novel marker of inflammation. Nevertheless, its potential utility in predicting the development of left ventricular aneurysm (LVA) remains unexplored. This study aims to investigate the association between MLR and the risk of LVA in patients presenting with acute ST segment elevation myocardial infarction (STEMI).

**Methods:**

A total of 551 patients were enrolled in the first cohort, and 471 patients were included in the validation cohort. To evaluate the predictive value of MLR for LVA, multivariable logistic regression analysis, restricted cubic splines (RCS) analysis, and receiver operating characteristic (ROC) analysis were employed.

**Results:**

The prevalence of LVA was 14.5% in the first cohort and 13.6% in the validation cohort. The multivariable logistic regression analysis revealed that individuals in the highest quartile of MLR (Q4) exhibited a significantly increased risk of LVA formation compared to those in the lowest quartile (Q1) in both cohorts (first cohort: OR = 3.07, 95% CI = 1.33–7.08, *P* = 0.009; validation cohort: OR = 3.55, 95% CI = 1.34–9.42, *P* = 0.011). The RCS analysis identified a positively nonlinear association in the first cohort and a positively linear association in the validation cohort between MLR and the risk of LVA (overall *P* < 0.05). Furthermore, the discriminative ability of MLR for LVA is 0.69 in the first cohort and 0.71 in the validation cohort, exceeding that of both monocyte and lymphocyte alone. The subgroup analyses further substantiated the robustness of our findings.

**Conclusion:**

An elevated MLR was independently linked to an increased risk of LVA development in patients with STEMI who received primary PCI. This readily available inflammatory index may offer supplementary prognostic information and could be considered for inclusion in future risk stratification models.

## Introduction

Left ventricular aneurysm (LVA), defined by the outward bulging of damaged myocardial tissue during both systolic and diastolic phases (1), represents a prevalent and severe complication of acute myocardial infarction (AMI) (2, 3). A growing amount of research suggests that LVA can increase the likelihood of arrhythmias (4), thromboembolic incidents (4), heart failure (5), and, in severe cases, cardiac rupture (6), all of which lead to a unfavorable prognosis for patients suffering from AMI (7). In light of the high incidence of LVA and its association with detrimental health outcomes (8), it has become critically important to identify effective predictors for the early detection and intervention of LVA.

Monocytes are integral components of the innate immune system, essential for maintaining immune homeostasis and responding to infections and inflammatory processes (9). In individuals with chronic kidney disease, an elevated monocyte count has been correlated with an increased risk of CVD events (10). A cohort study by Yamamoto et al. found that a high monocyte count serves as an independent and incremental predictor of cardiovascular events in patients with coronary artery disease (CAD) (11). In contrast, lymphocytes, which are pivotal to adaptive immune responses, play an opposing role to monocytes in the context of cardiovascular diseases—for example, a retrospective study demonstrated that a low lymphocyte count in patients with acute heart failure was associated with increased in-hospital mortality during the hospitalization period (12). Bian et al. found that a decreased percentage of lymphocytes could serve as an independent predictor for acute coronary syndrome upon admission and was associated with 1-year major adverse cardiac events during clinical follow-up in patients with coronary heart disease (13). The monocyte-to-lymphocyte ratio (MLR), a novel inflammatory marker, has garnered increasing attention in the field of cardiovascular diseases—for example, a retrospective analysis of 46,289 individuals revealed that an elevated MLR was correlated with an increased risk of cardiovascular disease (CVD), congestive heart failure, coronary artery disease, angina pectoris, heart attack, and stroke (14). Among patients with CAD and low-density lipoprotein cholesterol (LDL-C) levels below 1.4 mmol/L, a higher MLR was linked to an increased risk of both cardiovascular and all-cause mortality (15). Nonetheless, the association between MLR and LVA formation in patients experiencing acute STEMI has not yet been explored. In the present study, we aim to investigate the association between MLR and the risk of LVA formation in the Chinese population.

## Materials and methods

### Study subjects

**Figure 1:**
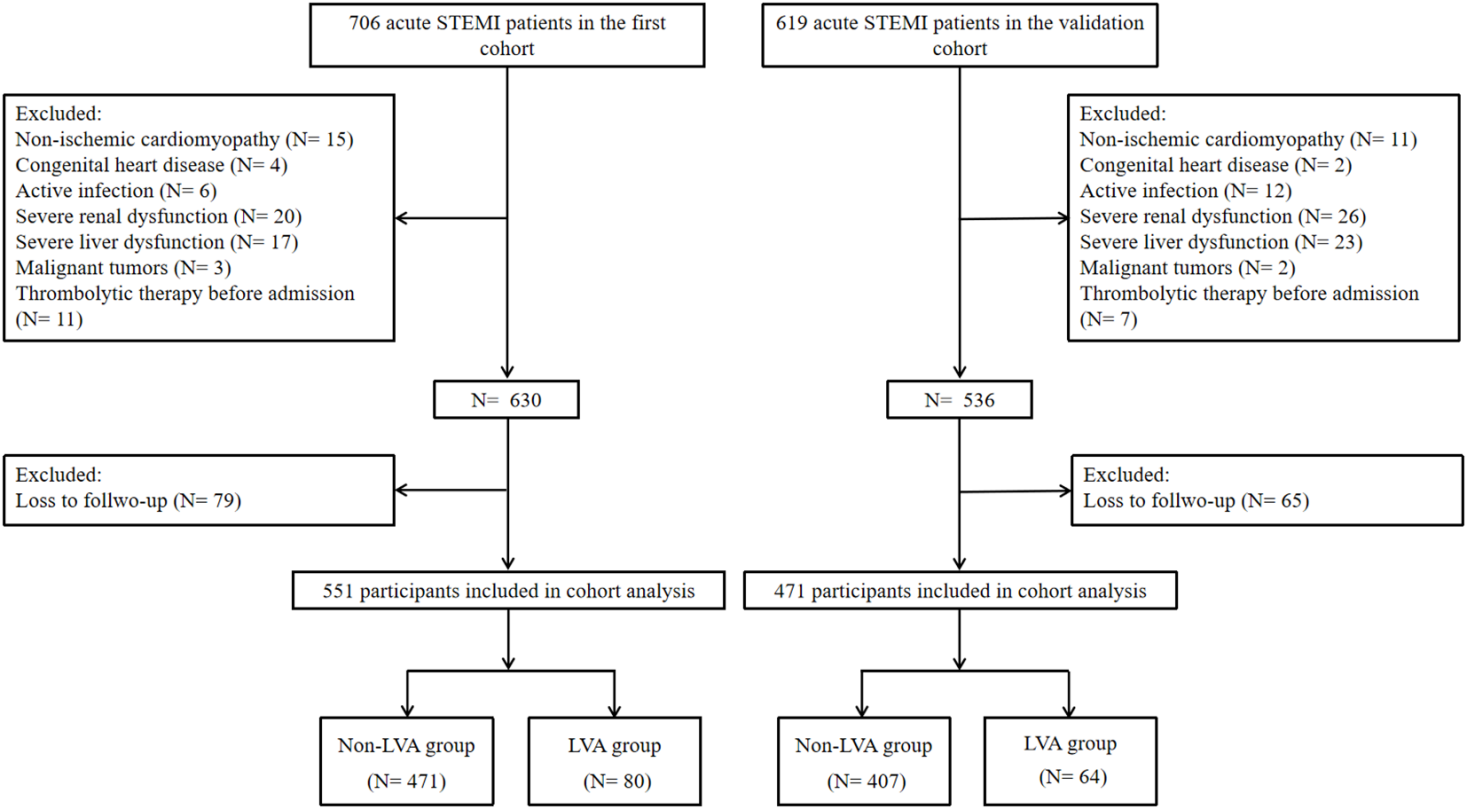
Flowchart of the patients’ enrollment. LVA, left ventricular aneurysm.

A cohort of 706 consecutive patients diagnosed with acute ST elevation myocardial infarction (STEMI) and treated with primary percutaneous coronary intervention (PCI) between December 2018 and February 2023 at the Central Hospital of Wuhan was enrolled in this study. The diagnosis of acute STEMI was established in accordance with the Fourth Universal Definition of Myocardial Infarction (16), which encompasses the following criteria: typical chest pain lasting over 30 min, with new ST segment elevation at the J point in at least two contiguous leads of >2 mm (0.2 mV) in men or >1.5 mm (0.15 mV) in women on admission electrocardiogram, and an increase in cardiac enzyme levels above the 99th percentile cutoff point for cardiac troponin I (cTnI). The criteria for exclusion included cases of non-ischemic cardiomyopathy (such as hypertrophic and dilated types), congenital heart defects, ongoing infections, kidney or liver failure, malignant neoplasms, individuals with a life expectancy of less than 1 year, those who received thrombolytic therapy before hospital admission, and patients lost to follow-up. Finally, the association analysis was conducted on a final sample of 551 patients (**Figure 1**). This study received approval from the Review Board of the Central Hospital of Wuhan and was conducted in accordance with the principles outlined in the Declaration of Helsinki. Written informed consents were obtained from all participants.

Furthermore, an independent cohort of 471 patients with acute STEMI who underwent primary PCI at Renmin Hospital of Wuhan University between July 2018 and June 2022 was consecutively enrolled to validate the predictive value of the MLR for LVA formation.

### Definitions

Upon admission, all patients underwent two-dimensional transthoracic echocardiography (TTE) and again at the conclusion of the 1st and 6th months of the follow-up period. The diagnosis of LVA was made through TTE following the protocol specified in the Coronary Artery Surgery Study (CASS) (17). The criteria for diagnosing LVA included (I) bulging of the left ventricular wall during diastole and systole, which showed either akinesia or dyskinesia, (II) a clear distinction of the infarcted segment, and (III) a lack of trabeculation in the involved segment. The diagnostic criteria for hypertension, diabetes mellitus, and smoking history have been previously documented (18). The Gensini score was computed utilizing the approach created by Celebi et al. (19).

### Laboratory analysis

Physicians who were unaware of the study’s objectives gathered demographic and clinical information utilizing the electronic medical records system. This data encompassed variables such as age, gender, hypertension, diabetes, smoking habits, left ventricular ejection fraction (LVEF), angiographic evaluation results, and medications prescribed at discharge. Fasting venous blood samples were systematically obtained from the elbow of all patients upon admission to the emergency department prior to primary percutaneous coronary intervention (PCI), as well as 8–12 h post-PCI. To assess the highest levels of cardiac enzymes, blood samples for troponin I (TnI) and lactate dehydrogenase (LDH) were collected, taken from a peripheral vein, every 12 h for the first 2 days and then every 24 h after that once the patient was admitted to the intensive coronary care unit.

### Statistical analysis

Continuous variables in the study were expressed either as the mean with standard deviation or as the median along with the interquartile range, depending on whether the data followed a normal distribution. Categorical variables were presented in terms of counts along with their respective percentages. To evaluate the differences between groups, one-way analyses of variance (for data with normal distribution), Kruskal–Wallis tests (for data that were not normally distributed), and chi-square tests (for categorical data) were utilized. The study population was classified into four distinct groups according to the quartiles of MLR. To investigate the relationship between MLR and the risk of LVA development, multivariate logistic regression analysis was performed. The association analyses were adjusted according to the following three models: model 1 was an unadjusted model, model 2 was adjusted for age and gender, and model 3 was further adjusted for hypertension, diabetes, smoking status, LAD (left anterior descending artery) as the Culprit vessel, the use of aspirin, clopidogrel/ticagrelor, statin, β-blockers, ACE inhibitors or ARB, and spironolactone, and Gensini score. The nonlinear relationship regarding MLR and the risk of LVA was assessed using restricted cubic splines (RCS) analysis. Analyses of subgroups were conducted to determine if the relationship between MLR and the risk of LVA varied according to factors such as gender, age, hypertension, diabetes, smoking status, LVEF (<50/≥50%), and LAD as the culprit vessel. To evaluate the predictive ability of different variables in forecasting LVA, receiver operating characteristic (ROC) curve analyses were also utilized.

The statistical evaluations for this research were performed utilizing SPSS version 26 (IBM Corporation, Armonk, NY, USA). All comparisons were assessed as two-sided, with the criterion for statistical significance established at *p <*0.05.

## Results

### Subjects’ characteristics

**Table 1** provides details regarding the baseline traits of the population involved. In the first cohort, the average age was 61.2 years (± 12.9), with female subjects constituting 19.78% of the patient cohort. During the follow-up period for TTE, 80 cases (14.5%) of LVA were identified. The group with LVA was characterized by older age and increased levels of LDH, D-dimer, C-reactive protein, Peak cTnI, N-terminal pro b-type natriuretic peptide (NT-proBNP), door-to-balloon time (DTB), Gensini score, and MLR (*P* < 0.05). Additionally, this group demonstrated a higher prevalence of spironolactone, angiotensin-converting enzyme inhibitors/angiotensin receptor blockers (ACEI/ARB), and thiazide/loop diuretics usage, alongside a greater incidence of the LAD being identified as the culprit vessel, compared to the non-LVA group (*P* < 0.05). Conversely, the LVA group exhibited a lower percentage of smokers and reduced levels of lymphocytes and left ventricular ejection fraction (LVEF) compared to those without LVA formation (*P* < 0.05).

**Table 1:** Baseline characteristics of the study population.

Characteristics	First cohort				Validation cohort			
	Whole cohort	Non-LVA patients	LVA patients	*P*-value	Whole cohort	Non-LVA patients	LVA patients	*P*-value
	*N* = 551	*N* = 471	*N* = 80		*N* = 471	*N* = 407	*N* = 64	
Demographics								
Gender, female (%)	109 (19.78)	87 (18.47)	22 (27.50)	0.061	76 (16.14)	59 (14.50)	17 (26.56)	0.015
Age (years)	61.2 ± 12.9	60.5 ± 12.8	65.1 ± 12.9	0.003	60.5 ± 12.2	60.1 ± 12.2	63.1 ± 12.1	0.067
Medical history, *n* (%)								
Hypertension	300 (54.45)	256 (54.35)	44 (55.00)	0.914	260 (55.20)	229 (56.27)	31 (48.44)	0.242
Diabetes	176 (31.94)	147 (31.21)	29 (36.25)	0.371	138 (29.30)	116 (28.50)	22 (34.38)	0.337
Smoking	298 (54.08)	263 (55.84)	35 (43.75)	0.045	269 (57.11)	237 (58.23)	32 (50.00)	0.216
Laboratory parameters								
WBC (×10^9^/L)	9.73 (7.77–12.13)	9.74 (7.81–12.05)	9.64 (7.70–12.43)	0.717	11.26 (9.51–13.83)	11.00 (9.38–13.73)	12.67 (10.45–14.41)	0.005
RBC (×10^9^/L)	4.47 ± 0.65	4.47 ± 0.66	4.42 ± 0.64	0.496	4.58 ± 0.61	4.61 ± 0.57	4.41 ± 0.76	0.05
Monocyte (×10^9^/L)	0.51 (0.37–0.70)	0.51 (0.37–0.68)	0.54 (0.36–0.76)	0.429	0.58 (0.40–0.85)	0.57 (0.40–0.80)	0.78 (0.46–1.08)	0.002
Lymphocyte (×10^9^/L)	1.34 (0.98–1.84)	1.38 (1.02–1.88)	1.15 (0.77–1.61)	0.003	1.07 (0.77–1.53)	1.08 (0.78–1.53)	0.98 (0.68–1.63)	0.264
Hemoglobin (g/L)	138.43 ± 21.40	139.09 ± 21.58	134.56 ± 19.98	0.08	140.72 ± 18.74	141.88 ± 17.75	133.38 ± 22.93	0.006
HbA1c (%)	6.00 (5.60–6.90)	6.00 (5.60–6.90)	6.10 (5.70–7.60)	0.18	6.10 (5.70–7.40)	6.10 (5.70–7.40)	6.05 (5.62–8.00)	0.915
ALT (U/L)	28 (18–48)	28 (18–47)	31 (18–54)	0.472	43 (27–63)	42 (26–63)	43 (31–67)	0.307
AST (U/L)	58 (25–160)	58 (25–154)	68 (26–232)	0.152	135 (69–257)	134 (68–251)	186 (80–335)	0.104
LDH (U/L)	312 (202–572)	284 (199–534)	471 (244–740)	<0.001	458 (295–685)	444 (281–656)	654 (397–884)	<0.001
Uric acid (umol/L)	371 (308–438)	373 (308–439)	352 (309–425)	0.323	373 (312–446)	370 (311–437)	401 (314–477)	0.102
TC (mmol/L)	4.70 ± 1.18	4.67 ± 1.18	4.83 ± 1.14	0.255	4.49 ± 1.04	4.49 ± 1.03	4.54 ± 1.14	0.68
HDL (mmol/L)	0.98 (0.84–1.14)	0.98 (0.83–1.13)	0.98 (0.84–1.19)	0.598	0.99 (0.84–1.19)	1.00 (0.85–1.19)	0.94 (0.80–1.17)	0.269
LDL (mmol/L)	3.12 ± 1.08	3.09 ± 1.07	3.30 ± 1.10	0.106	2.94 ± 0.97	2.94 ± 0.96	2.92 ± 0.98	0.891
D-dimer (ug/mL)	0.38 (0.23–0.65)	0.36 (0.22–0.61)	0.50 (0.29–0.99)	0.002	0.39 (0.21–0.81)	0.35 (0.20–0.69)	0.83 (0.40–2.11)	<0.001
C-reactive protein (mg/L)	0.70 (0.23–1.93)	0.60 (0.22–1.64)	1.98 (0.40–4.08)	<0.001	2.57 (0.84–7.36)	2.40 (0.84–6.10)	6.70 (1.38–29.61)	<0.001
Peak cTnI (ng/mL)	19.7 (3.6–50.0)	16.2 (3.5– 48.1)	26.8 (5.0–50.0)	0.041	21.2 (7.1–39.0)	19.5 (6.5–39.5)	32.0 (32.0–38.7)	<0.001
NT-proBNP (pg/mL)	586 (213–1,722)	481 (201–1,317)	3,336 (952–7,633)	<0.001	362 (113–1,433)	314 (103–1,116)	2,287 (250–5,133)	<0.001
MLR	0.36 (0.25–0.54)	0.35 (0.24–0.51)	0.49 (0.28–0.67)	<0.001	0.52 (0.36–0.77)	0.50 (0.35–0.74)	0.67 (0.51–0.97)	<0.001
LVEF (%)	54.15 ± 8.52	56.00 ± 7.09	43.24 ± 8.08	<0.001	48.96 ± 7.77	50.53 ± 6.80	39.76 ± 6.64	<0.001
Medication at hospital discharge, *n* (%)								
Statin	551 (100.0)	471 (100.0)	80 (100.0)	>0.99	471 (100.0)	407 (100.0)	64 (100.0)	>0.99
Aspirin	551 (100.0)	471 (100.0)	80 (100.0)	>0.99	471 (100.0)	407 (100.0)	64 (100.0)	>0.99
Clopidogrel/ticagrelor	551 (100.0)	471 (100.0)	80 (100.0)	>0.99	471 (100.0)	407 (100.0)	64 (100.0)	>0.99
Beta blocker	474 (86.03)	407 (86.41)	67 (83.75)	0.526	415 (88.11)	362 (88.94)	53 (82.81)	0.159
Spironolactone	198 (35.93)	143 (30.36)	55 (68.75)	<0.001	160 (33.97)	114 (28.01)	46 (71.88)	<0.001
ACEI/ARB	405 (73.50)	336 (71.34)	69 (86.25)	0.005	365 (77.49)	308 (75.68)	57 (89.06)	0.017
Thiazide/loop diuretic	199 (36.12)	145 (30.79)	54 (67.50)	<0.001	162 (34.39)	114 (28.01)	48 (75.00)	<0.001
Coronary artery injury								
Culprit artery—LAD	289 (52.45)	221 (46.92)	68 (85.00)	<0.001	283 (60.08)	224 (55.04)	59 (92.19)	<0.001
Proximal	188 (65.05)	145 (65.61)	43 (63.24)	0.807	210 (74.20)	163 (72.77)	47 (79.66)	0.55
Mid	88 (30.45)	67 (30.32)	21 (30.88)		69 (24.38)	57 (25.45)	12 (20.34)	
Distal	13 (4.50)	9 (4.07)	4 (5.88)		4 (1.41)	4 (1.79)	0 (0.00)	
Culprit artery—LCX	62 (11.25)	60 (12.74)	2 (2.50)	<0.001	54 (11.46)	52 (12.78)	2 (3.12)	<0.001
Proximal	26 (41.94)	25 (41.67)	1 (50.00)	>0.99	23 (42.59)	23 (44.23)	0 (0.00)	0.18
Mid	31 (50.00)	30 (50.00)	1 (50.00)		26 (48.15)	25 (48.08)	1 (50.00)	
Distal	5 (8.06)	5 (8.33)	0 (0.00)		5 (9.26)	4 (7.69)	1 (50.00)	
Culprit artery—RCA	200 (36.30)	190 (40.34)	10 (12.50)	<0.001	134 (28.45)	131 (32.19)	3 (4.69)	<0.001
Proximal	87 (43.50)	81 (42.63)	6 (60.00)	0.44	75 (55.97)	74 (56.49)	1 (33.33)	0.34
Mid	87 (43.50)	83 (43.68)	4 (40.00)		44 (32.84)	43 (32.82)	1 (33.33)	
Distal	26 (13.00)	26 (13.68)	0 (0.00)		15 (11.19)	14 (10.69)	1 (33.33)	
Post-procedural TIMI flow <3	27 (4.90)	21 (4.46)	6 (7.50)	0.376	12 (2.55)	10 (2.46)	2 (3.12)	>0.99
Use of drug-eluting stent	505 (91.65)	431 (91.51)	74 (92.50)	0.767	449 (95.33)	388 (95.33)	61 (95.31)	>0.99
Balloon dilation	547 (99.27)	468 (99.36)	79 (98.75)	0.467	463 (98.30)	400 (98.28)	63 (98.44)	>0.99
DTB, min	68 (50–86)	67 (48–85)	77 (59–92)	0.01	64 (41–85)	62(39–84)	79 (50–96)	<0.001
Gensini score	72 (47–92)	65 (44–89)	88 (64–106)	<0.001	64 (40–82)	60 (40–80)	80 (48–96)	<0.001

WBC, white blood cell count; RBC, red blood cell count; HbA1c, glycated hemoglobin; ALT, alanine aminotransferase; AST, aspartate aminotransferase; LDH, lactate dehydrogenase; TC, total cholesterol; HDL; high-density lipoprotein; LDL, low-density lipoprotein; NT-proBNP, N-terminal pro b-type natriuretic peptide; MLR, monocyte-to-lymphocyte ratio; LVEF, left ventricular ejection fraction; ACEI/ARB, angiotensin-converting enzyme inhibitor/angiotensin receptor blocker; LAD, left anterior descending artery; LCX, left circumflex artery; RCA, right coronary artery; DTB, door-to-balloon time; LVA, left ventricular aneurysm.

In the validation cohort, the mean age was 60.5 years (± 12.2), with female subjects comprising 16.14% of the patient population. During the TTE follow-up, 64 cases (13.6%) of LVA were identified. Patients in the LVA group demonstrated a higher likelihood of being female and exhibited significantly elevated levels of WBC, monocyte, LDH, D-dimer, C-reactive protein, Peak cTnI, NT-proBNP, DTB, Gensini score, and MLR. Additionally, there was a higher prevalence of spironolactone, ACEI/ARB, and thiazide/loop diuretics usage as well as a greater incidence of LAD as the culprit vessel compared to the non-LVA group (*P* < 0.05). Conversely, the levels of hemoglobin and LVEF were lower in patients with LVA versus those without it (*P* < 0.05).

### Baseline characteristics based on the quantiles of MLR

**Table 2** delineates the baseline characteristics of participants stratified by MLR quartiles within the first cohort. The incidence rates of LVA across quartiles Q1, Q2, Q3, and Q4 were 8.7%, 10.87%, 14.49%, and 24.09%, respectively. The participants in the highest MLR quartile (Q4) exhibited elevated levels of WBC, monocyte, aspartate aminotransferase (AST), LDH, high-density lipoprotein (HDL), D-dimer, C-reactive protein, peak cTnI, NT-proBNP, and Gensini score. Additionally, there was a higher prevalence of hypertension in this group compared to those in the lower MLR quartiles. Conversely, the levels of lymphocyte and LVEF were significantly reduced in participants within MLR quartile Q4 compared to those in the other quartiles (*P* < 0.05).

**Table 2:** Characteristics of the first cohort according to the quartiles of MLR.

Characteristics	MLR				*P*-value
	Q1 (<0.25)	Q2 (≥0.25, <0.36)	Q3 (≥0.36, <0.54)	Q4 (≥0.54)	
Participants, number	138	138	138	137	
Demographics					
Gender, female (%)	35 (25.36)	27 (19.57)	25 (18.12)	22 (16.06)	0.246
Age (years)	60.4 ± 12.0	59.3 ± 13.0	62.6 ± 12.9	62.3 ± 13.3	0.097
Medical history, *n* (%)					
Hypertension	60 (43.48)	77 (55.80)	76 (55.07)	87 (63.50)	0.01
Diabetes	43 (31.16)	41 (29.71)	51 (36.96)	41 (29.93)	0.531
Smoking	79 (57.25)	72 (52.17)	70 (50.72)	77 (56.20)	0.652
Laboratory parameters					
WBC (×10^9^/L)	8.48 (7.22–10.83)	9.39 (7.82–11.41)	9.72 (8.13–11.91)	11.26 (8.92–14.71)	<0.001
RBC (×10^9^/L)	4.53 ± 0.57	4.51 ± 0.57	4.41 ± 0.73	4.41 ± 0.73	0.25
Monocyte (×10^9^/L)	0.40 (0.29–0.50)	0.47 (0.35–0.58)	0.53 (0.42–0.66)	0.75 (0.59–0.98)	<0.001
Lymphocyte (×10^9^/L)	2.02 (1.50–2.86)	1.50 (1.16–1.93)	1.20 (0.92–1.52)	0.98 (0.79–1.30)	<0.001
Hemoglobin (g/L)	140.55 ± 18.38	140.08 ± 19.29	137.99 ± 25.43	135.09 ± 21.57	0.134
HbA1c, (%)	6.20 (5.73, 6.90)	6.00 (5.60, 6.90)	5.90 (5.50, 6.95)	6.00 (5.60, 6.90)	0.207
ALT (U/L)	23 (17–39)	27 (19–37)	33 (19–58)	32 (18–55)	0.004
AST (U/L)	37 (20–83)	50 (23–134)	75 (32–233)	108 (36–250)	<0.001
LDH (U/L)	208 (172–300)	276 (200–453)	358 (226–691)	491 (311–824)	<0.001
Uric acid (umol/L)	363 (314–422)	372 (298–425)	361 (304–447)	389 (315–456)	0.417
TC (mmol/L)	4.84 ± 1.17	4.71 ± 1.09	4.59 ± 1.00	4.603 ± 1.41	0.326
HDL (mmol/L)	1.00 (0.82–1.13)	0.94 (0.82-1.06)	1.00 (0.89-1.15)	1.02 (0.82-1.22)	0.039
LDL (mmol/L)	3.27 ± 1.11	3.13 ± 1.01	2.95 ± 0.85	3.12 ± 1.28	0.099
D-dimer (ug/mL)	0.36 (0.24–0.54)	0.34 (0.21–0.55)	0.38 (0.23–0.65)	0.45 (0.26–0.91)	0.012
C-reactive protein (mg/L)	0.52 (0.21–1.26)	0.61 (0.23–1.81)	0.52 (0.20–1.65)	1.23 (0.40–3.73)	<0.001
Peak cTnI (ng/mL)	4.43 (0.72–26.22)	17.84 (3.60–40.54)	26.15 (4.80–50.00)	30.00 (10.93–50.00)	<0.001
NT-proBNP (pg/mL)	346 (127–876)	403 (200–1,460)	521 (279–1,468)	1,305 (546–2,964)	<0.001
LVEF (%)	55.17 ± 9.00	55.85 ± 7.83	53.75 ± 7.79	51.79 ± 8.90	<0.001
Medication at hospital discharge, *n* (%)					
Statin	138 (100.0)	138 (100.0)	138 (100.0)	137 (100.0)	>0.99
Aspirin	138 (100.0)	138 (100.0)	138 (100.0)	137 (100.0)	>0.99
Clopidogrel/ticagrelor	138 (100.0)	138 (100.0)	138 (100.0)	137 (100.0)	>0.99
Beta blocker	121 (87.68)	118 (85.51)	116 (84.06)	119 (86.86)	0.833
Spironolactone	49 (35.51)	40 (28.99)	55 (39.86)	54 (39.42)	0.208
ACEI/ARB	107 (77.54)	98 (71.01)	96 (69.57)	104 (75.91)	0.377
Thiazide/loop diuretic	49 (35.51)	39 (28.26)	55 (39.86)	56 (40.88)	0.117
Coronary artery injure					
Culprit artery—LAD	65 (47.10)	66 (47.83)	80 (57.97)	78 (56.93)	0.362
Proximal	44 (67.69)	39 (59.09)	57 (71.25)	48 (61.54)	0.696
Mid	18 (27.69)	24 (36.36)	21 (26.25)	25 (32.05)	
Distal	3 (4.62)	3 (4.55)	2 (2.50)	5 (6.41)	
Culprit artery—LCX	16 (11.59)	15 (10.87)	16 (11.59)	15 (10.95)	0.362
Proximal	8 (50.00)	5 (33.33)	6 (37.50)	7 (46.67)	0.848
Mid	8 (50.00)	8 (53.33)	8 (50.00)	7 (46.67)	
Distal	0 (0.00)	2 (13.33)	2 (12.50)	1 (6.67)	
Culprit artery—RCA	57 (41.30)	57 (41.30)	42 (30.43)	44 (32.12)	0.362
Proximal	24 (42.11)	25 (43.86)	18 (42.86)	20 (45.45)	0.744
Mid	27 (47.37)	21 (36.84)	20 (47.62)	19 (43.18)	
Distal	6 (10.53)	11 (19.30)	4 (9.52)	5 (11.36)	
Post-procedural TIMI flow <3	12 (8.70)	4 (2.90)	5 (3.62)	6 (4.38)	0.111
Use of drug-eluting stent	128 (92.75)	130 (94.20)	124 (89.86)	123 (89.78)	0.457
Balloon dilation	137 (99.28)	137 (99.28)	138 (100.00)	135 (98.54)	0.481
DTB, min	65.00 (47.25–84.75)	66.00 (47.00–87.75)	68.50 (51.00–85.75)	71.00 (55.00–86.00)	0.202
Gensini score	62 (40–93)	66 (46–89)	76 (50–92)	80 (56–98)	0.026
LVA (%)	12 (8.70)	15 (10.87)	20 (14.49)	33 (24.09)	0.002

WBC, white blood cell count; RBC, red blood cell count; HbA1c, glycated hemoglobin; ALT, alanine aminotransferase; AST, aspartate aminotransferase; LDH, lactate dehydrogenase; TC, total cholesterol; HDL; high-density lipoprotein; LDL, low-density lipoprotein; NT-proBNP, N-terminal pro b-type natriuretic peptide; MLR, monocyte-to-lymphocyte ratio; LVEF, left ventricular ejection fraction; ACEI/ARB, angiotensin-converting enzyme inhibitor/angiotensin receptor blocker; LAD, left anterior descending artery; LCX, left circumflex artery; RCA, right coronary artery; DTB, door-to-balloon time; LVA, left ventricular aneurysm.

In the validation cohort, the incidence rates of LVA across quartiles Q1, Q2, Q3, and Q4 were 5.98%, 10.17%, 14.41%, and 23.73%, respectively (**Table 3**). The participants in the highest MLR quartile (Q4) were older and exhibited significantly elevated levels of WBC, monocyte, alanine aminotransferase (ALT), AST, LDH, HDL, D-dimer, C-reactive protein, Peak cTnI, and NT-proBNP, along with increased utilization of spironolactone and thiazide/loop diuretics (*P* < 0.05). In contrast, the participants in MLR quartile Q4 demonstrated significantly lower levels of red blood cell count, lymphocyte, hemoglobin, and LVEF compared to those in the other quartiles (*P* < 0.05).

**Table 3:** Characteristics of the validation cohort according to the quartiles of MLR.

Characteristics	MLR				*P*-value
	Q1 (<0.36)	Q2 (≥0.36, <0.52)	Q3 (≥0.52, <0.77)	Q4 (≥0.77)	
Participants, number	117	118	118	118	
Demographics					
Gender, female (%)	20 (17.09)	16 (13.56)	22 (18.64)	18 (15.25)	0.735
Age (years)	57.6 ± 10.8	60.4 ± 10.6	60.7 ± 12.8	63.3 ± 13.7	0.004
Medical history, *n* (%)					
Hypertension	64 (54.70)	65 (55.08)	65 (55.08)	66 (55.93)	0.998
Diabetes	31 (26.50)	33 (27.97)	36 (30.51)	38 (32.20)	0.775
Smoking	71 (60.68)	71 (60.17)	65 (55.08)	62 (52.54)	0.52
Laboratory parameters					
WBC (×10^9^/L)	10.13 (8.71–12.05)	10.61 (9.32–12.91)	11.27 (9.61–13.87)	13.65 (10.78–15.82)	<0.001
RBC (×10^9^/L)	4.71 ± 0.59	4.63 ± 0.51	4.54 ± 0.61	4.46 ± 0.69	0.009
Monocyte (×10^9^/L)	0.38 (0.27–0.55)	0.49 (0.37–0.68)	0.64 (0.51–0.90)	0.91 (0.68–1.27)	<0.001
Lymphocyte (×10^9^/L)	1.45 (1.02–1.90)	1.11 (0.83–1.52)	1.04 (0.78–1.38)	0.80 (0.59–1.07)	<0.001
Hemoglobin (g/L)	144.23 ± 16.69	141.99 ± 16.66	138.78 ± 18.43	137.92 ± 22.18	0.035
HbA1c (%)	6.30 (5.90–7.80)	6.20 (5.70–6.77)	5.90 (5.70–7.30)	5.95 (5.50–7.10)	0.05
ALT (U/L)	38 (28–54)	38 (25–57)	46 (29–64)	51 (29–75)	0.015
AST (U/L)	102 (48–211)	135 (58–248)	150 (84–287)	200 (100–324)	<0.001
LDH (U/L)	349 (244–523)	447 (256–614)	466 (326–737)	653 (397–918)	<0.001
Uric acid (umol/L)	361 (289–426)	363 (312–445)	374 (312–433)	381 (327–470)	0.065
TC (mmol/L)	4.54 ± 0.99	4.35 ± 1.02	4.61 ± 1.01	4.47 ± 1.13	0.253
HDL (mmol/L)	0.93 (0.77–1.15)	0.99 (0.83–1.17)	1.00 (0.87–1.20)	1.04 (0.85–1.26)	0.013
LDL (mmol/L)	2.97 ± 0.91	2.88 ± 0.88	3.02 ± 0.95	2.88 ± 1.10	0.597
D-dimer (ug/mL)	0.30 (0.17–0.47)	0.34 (0.20–0.68)	0.42 (0.26–0.88)	0.65 (0.27–1.77)	<0.001
C-reactive protein (mg/L)	1.46 (0.70–3.76)	2.25 (0.57–5.91)	3.06 (0.86–7.42)	5.31 (1.93–21.03)	<0.001
Peak cTnI (ng/mL)	14.35 (4.29–32.75)	23.00 (6.68–40.21)	23.00 (9.31–39.50)	28.00 (9.88–42.37)	0.004
NT-proBNP (pg/mL)	234 (65–771)	250 (102–647)	395 (129–1,514)	1,537 (278–5,032)	<0.001
LVEF (%)	51.74 ± 7.52	49.92 ± 7.21	48.27 ± 7.18	45.99 ± 8.00	<0.001
Medication at hospital discharge, *n* (%)					
Statin	117 (100.0)	118 (100.0)	118 (100.0)	118 (100.0)	>0.99
Aspirin	117 (100.0)	118 (100.0)	118 (100.0)	118 (100.0)	>0.99
Clopidogrel/ticagrelor	117 (100.0)	118 (100.0)	118 (100.0)	118 (100.0)	>0.99
Beta blocker	102 (87.18)	102 (86.44)	105 (88.98)	106 (89.83)	0.842
Spironolactone	29 (24.79)	31 (26.27)	40 (33.90)	60 (50.85)	<0.001
ACEI/ARB	88 (75.21)	92 (77.97)	94 (79.66)	91 (77.12)	0.875
Thiazide/loop diuretic	29 (24.79)	29 (24.58)	42 (35.59)	62 (52.54)	<0.001
Coronary artery injury					
Culprit artery—LAD	67 (57.26)	72 (61.02)	68 (57.63)	76 (64.41)	0.36
Proximal	56 (83.58)	48 (66.67)	46 (67.65)	60 (78.95)	0.05
Mid	11 (16.42)	21 (29.17)	22 (32.35)	15 (19.74)	
Distal	0 (0.00)	3 (4.17)	0 (0.00)	1 (1.32)	
Culprit artery—LCX	16 (13.68)	18 (15.25)	12 (10.17)	8 (6.78)	0.36
Proximal	4 (25.00)	8 (44.44)	7 (58.33)	4 (50.00)	0.414
Mid	10 (62.50)	9 (50.00)	3 (25.00)	4 (50.00)	
Distal	2 (12.50)	1 (5.56)	2 (16.67)	0 (0.00)	
Culprit artery—RCA	34 (29.06)	28 (23.73)	38 (32.20)	34 (28.81)	0.36
Proximal	21 (61.76)	15 (53.57)	18 (47.37)	21 (61.76)	0.46
Mid	7 (20.59)	11 (39.29)	16 (42.11)	10 (29.41)	
Distal	6 (17.65)	2 (7.14)	4 (10.53)	3 (8.82)	
Post-procedural TIMI Fow <3	1 (0.85)	4 (3.39)	2 (1.69)	5 (4.24)	0.396
Use of drug-eluting stent	111 (94.87)	115 (97.46)	111 (94.07)	112 (94.92)	0.632
Balloon dilation	116 (99.15)	114 (96.61)	116 (98.31)	117 (99.15)	0.544
DTB, min	63 (38–83)	60 (39–84)	63 (41–86)	69 (45–92)	0.432
Gensini score	52 (40–82)	64 (41–83)	57 (40–80)	72 (42–85)	0.174
LVA (%)	7 (5.98)	12 (10.17)	17 (14.41)	28 (23.73)	<0.001

WBC, white blood cell count; RBC, red blood cell count; HbA1c, glycated hemoglobin; ALT, alanine aminotransferase; AST, aspartate aminotransferase; LDH, lactate dehydrogenase; TC, total cholesterol; HDL; high-density lipoprotein; LDL, low-density lipoprotein; NT-proBNP, N-terminal pro b-type natriuretic peptide; MLR, monocyte-to-lymphocyte ratio; LVEF, left ventricular ejection fraction; ACEI/ARB, angiotensin-converting enzyme inhibitor/angiotensin receptor blocker; LAD, left anterior descending artery; LCX, left circumflex artery; RCA, right coronary artery; DTB, door-to-balloon time; LVA, left ventricular aneurysm.

### Correlation of MLR with LVA formation

**Table 4** presents a detailed analysis of the association between MLR quantiles and the risk of LVA as assessed through logistic regression analysis. In the first cohort, the patients in the fourth quartile (Q4) exhibited a significantly increased risk of LVA compared to those in the first quartile (Q1) across all models: model 1 (OR = 3.33, 95% CI = 1.64–6.78, *P* < 0.001), model 2 (OR = 3.41, 95% CI = 1.65–7.03, *P* < 0.001), and model 3 (OR = 3.07, 95% CI = 1.33–7.08, *P* = 0.009). In the validation cohort, similar findings were observed, with the participants in MLR Q4 demonstrating an elevated risk of LVA formation, both without and with adjustment for confounding variables: model 1 (OR = 4.89, 95% CI = 2.04–11.71, *P* < 0.001) and model 3 (OR = 3.55, 95% CI = 1.34–9.42, *P* = 0.011).

**Table 4:** Logistic regression analysis results for the association between MLR and the risk of LVA.

Cohorts	LVA	Model 1		Model 2		Model 3	
		OR (95% CI)	*P*-value	OR (95% CI)	*P*-value	OR (95% CI)	*P*-value
First cohort	MLR, categories						
	Q1	Reference		Reference		Reference	
	Q2	1.28 (0.58–2.85)	0.544	1.35 (0.60–3.03)	0.462	1.56 (0.62–3.93)	0.343
	Q3	1.78 (0.83–3.80)	0.136	1.76 (0.81–3.79)	0.15	1.40 (0.58–3.37)	0.456
	Q4	3.33 (1.64–6.78)	<0.001	3.41 (1.65–7.03)	<0.001	3.07 (1.33–7.08)	0.009
Validation cohort	MLR, categories						
	Q1	Reference		Reference		Reference	
	Q2	1.78 (0.67–4.69)	0.244	1.82 (0.68–4.83)	0.23	1.77 (0.61–5.08)	0.29
	Q3	2.64 (1.05–6.64)	0.038	2.58 (1.02–6.53)	0.045	2.46 (0.89–6.74)	0.081
	Q4	4.89 (2.04–11.71)	<0.001	4.90 (2.01–11.93)	<0.001	3.55 (1.34–9.42)	0.011

OR, odds ratio; CI, confidence interval; Model 1, unadjusted; Model 2, adjusted for age and gender; Model 3, model 2 + further adjusted for hypertension, diabetes, smoking status, LAD as the culprit vessel, use of aspirin, clopidogrel/ticagrelor, statin, β-blockers, ACE inhibitors or ARB, and spironolactone, and Gensini score.

**Figure 2:**
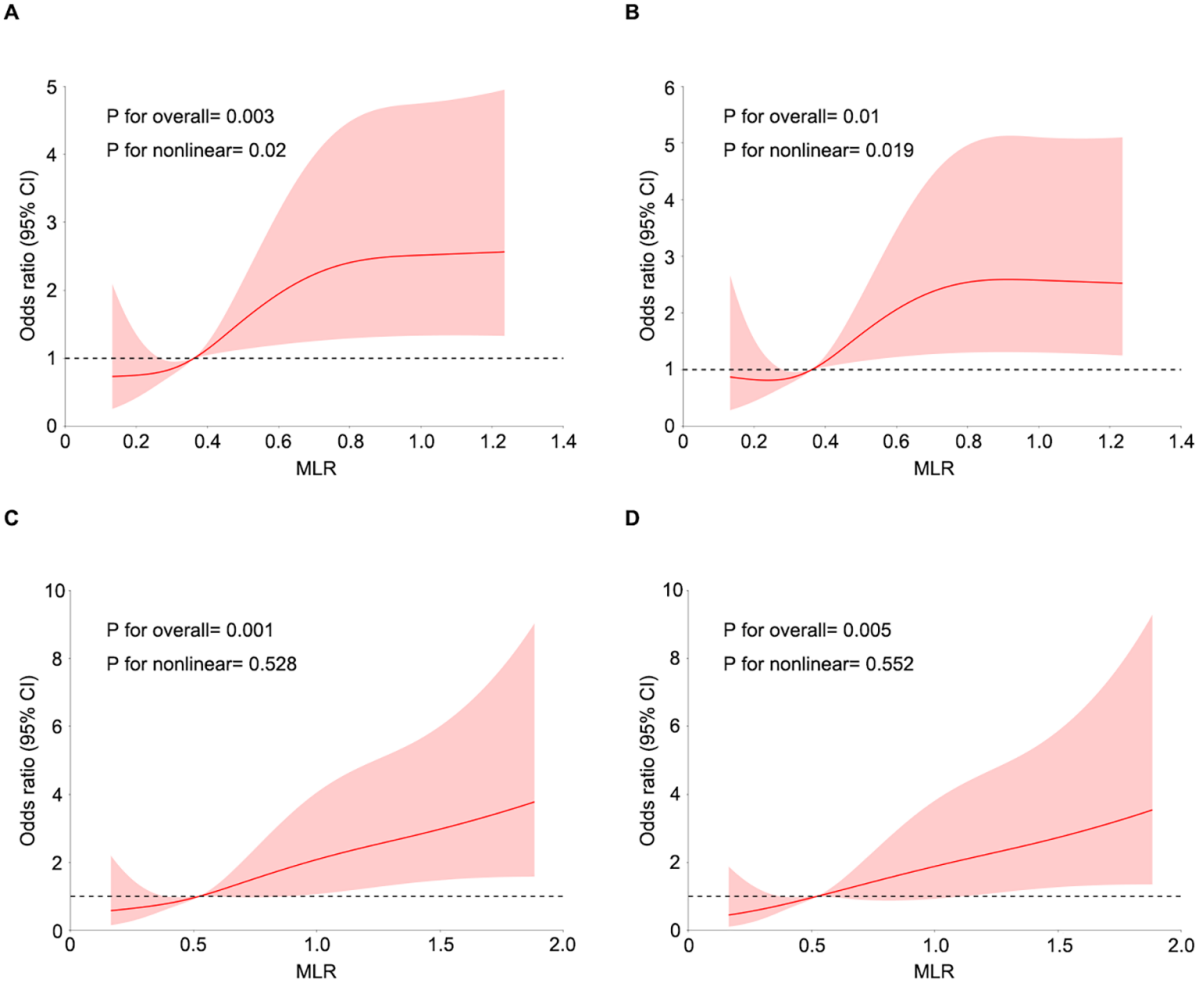
Restricted cubic spline curves for LVA by MLR using logistic regression analysis without **(A, C)** or with **(B, D)** adjustment for other covariates in the first **(A, B)** and validation cohorts (**C**, **D**).

We utilized a four-knot restricted cubic spline (RCS) regression model to effectively elucidate the dose–response relationship between the MLR and the risk of LVA development. **Figure 2A** illustrates a positive nonlinear association between MLR and the risk of LVA development without adjustment for any covariates in the first cohort (nonlinear *P* < 0.05). After adjusting for potential confounding variables—including age, gender, hypertension, diabetes, smoking status, LAD as the culprit vessel, the use of β-blockers, ACE inhibitors or ARB, spironolactone, and clopidogrel/ticagrelor, and Gensini score—a consistent positive nonlinear relationship between MLR and the risk of LVA development was observed (**Figure 2B**). In the validation cohort, a positive linear association was identified between MLR and the risk of LVA development without or with adjustment for other confounders (**Figures 2C, D**).

### Relationship between MLR and clinical parameters

**Figure 3:**
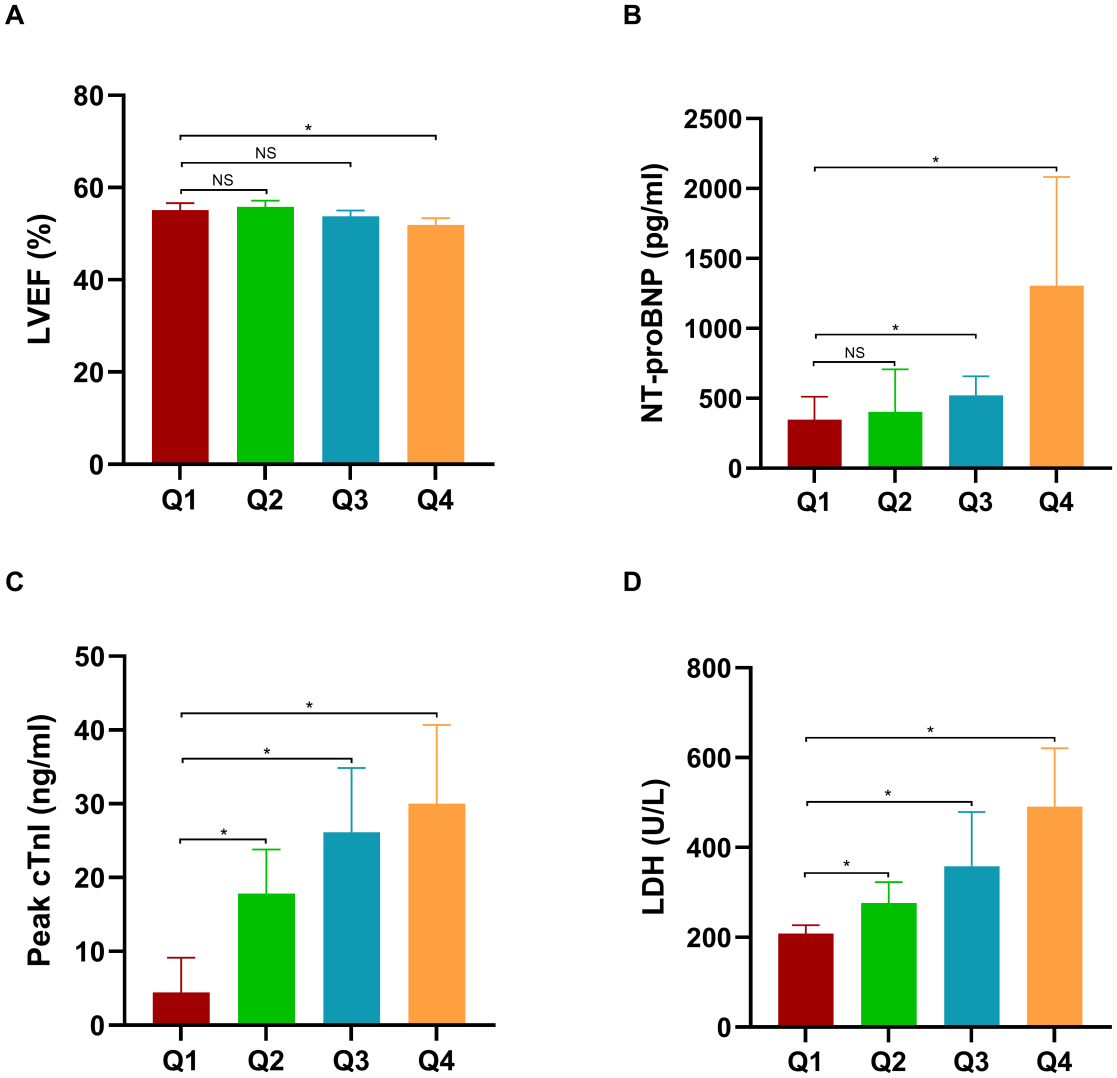
Comparison of LVEF, NT-proBNP, Peak cTnI, and LDH across different MLR groups in the first cohort. LVEF, left ventricular ejection fraction; NT-proBNP, N-terminal pro b-type natriuretic peptide; LDH, lactate dehydrogenase.

**Figure 4:**
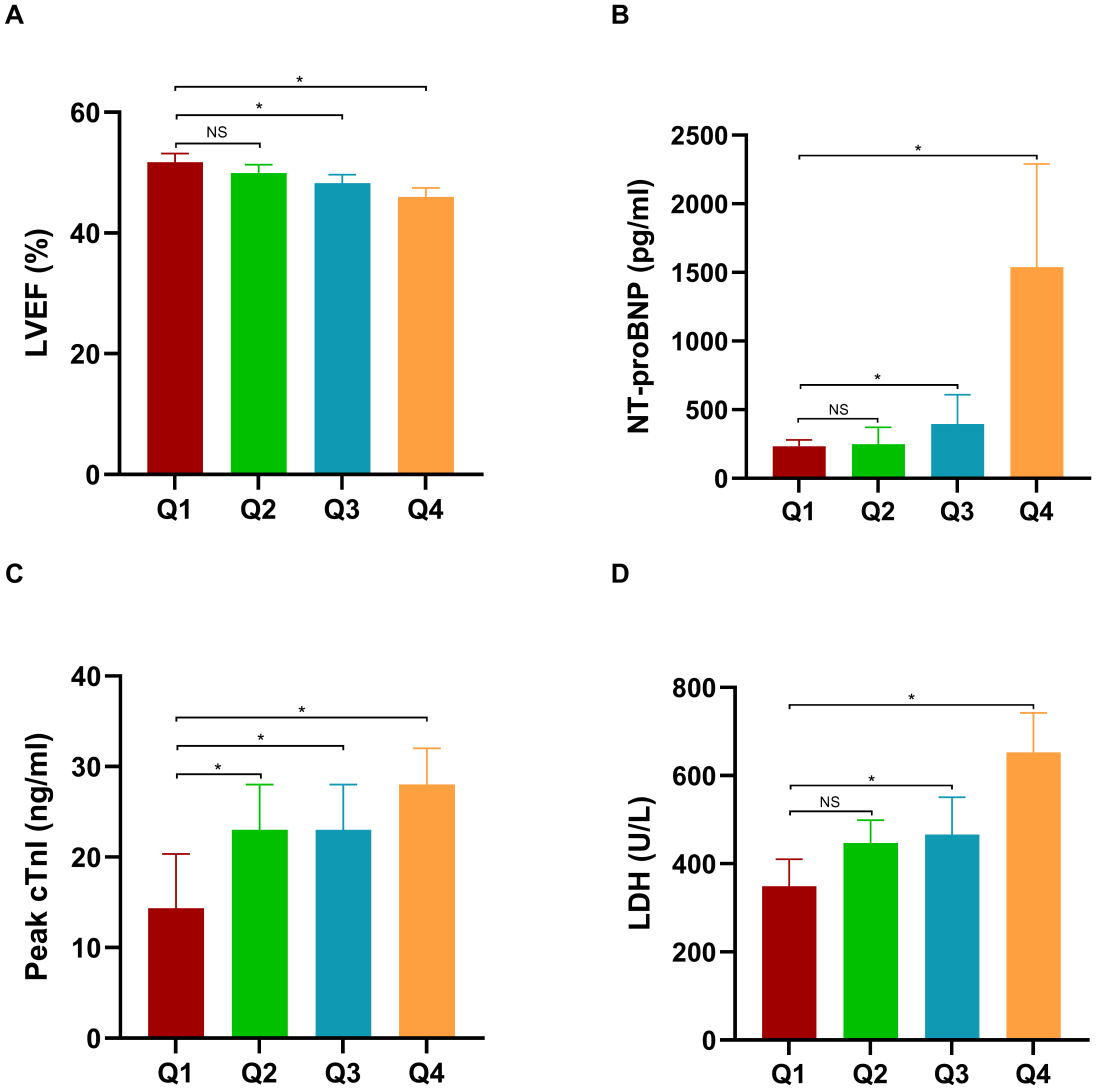
Comparison of LVEF, NT-proBNP, Peak cTnI, and LDH across different MLR groups in the validation cohort. LVEF, left ventricular ejection fraction; NT-proBNP, N-terminal pro b-type natriuretic peptide; LDH, lactate dehydrogenase.

To evaluate the relationship between MLR and myocardial injury and cardiac function, LVEF, NT-proBNP, Peak cTnI, and LDH were compared across different MLR groups. As depicted in **Figures 3**, **4**, individuals in the third (Q3) and fourth (Q4) quartiles exhibited significantly higher levels of NT-proBNP, peak cTnI, and LDH compared to those in the first quartile (Q1) in both cohorts (*P* < 0.05). Additionally, in the first cohort, the Peak cTnI level was elevated in the second quartile (Q2) relative to the Q1 group. In the validation cohort, both peak cTnI and LDH levels were higher in the Q2 group compared to the Q1 group. In contrast, LVEF was significantly reduced in individuals in the Q4 quartile compared to those in the Q1 quartile across both cohorts (*P* < 0.05).

### Discriminative power analysis

**Figure 5:**
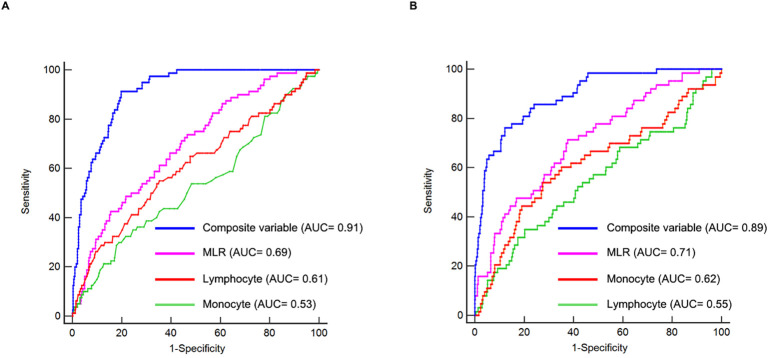
Receiver operating characteristic curves for the prediction of LVA formation in the first **(A)** and validation cohorts **(B)**. AUC, area under the curve.

In order to evaluate and compare the predictive capabilities of monocyte, lymphocyte, MLR, and the composite variable (which integrates the MLR with factors including age, gender, hypertension, diabetes, smoking status, LAD as the culprit vessel, and LVEF), we conducted ROC analysis. In the first cohort, the mean AUCs for monocyte, lymphocyte, MLR, and the composite variable were 0.53 (0.49–0.57), 0.61 (0.56–0.65), 0.69 (0.65–0.73), and 0.91 (0.88–0.93), respectively (**Table 5**). In the validation cohort, the average AUCs for monocyte, lymphocyte, MLR, and the composite variable were 0.62 (0.57–0.66), 0.55 (0.50–0.60), 0.71 (0.66–0.75), and 0.89 (0.86–0.92), respectively (**Table 5**). Notably, MLR demonstrated significantly higher AUCs for predicting the risk of LVA formation compared to monocyte (*P* < 0.05) and lymphocyte (*P* < 0.05) in both cohorts (**Table 5**, **Figure 5**). Moreover, the composite variable exhibited the most robust predictive capability (*P* < 0.001). Additionally, the best cutoff values of MLR were 0.128 in the first cohort and 0.117 in the validation cohort.

**Table 5:** Analysis of the ROC curve for predictive power of left ventricular aneurysm formation.

Cohort	Variables	AUC	95% CI	Specificity	Sensitivity	*p*-value^a^
First cohort	MLR	0.69	0.65–0.73	0.59	0.74	–
	Monocyte	0.53	0.49–0.57	0.81	0.3	0.001
	Lymphocyte	0.61	0.56–0.65	0.35	0.45	0.027
	Composite variable	0.91	0.88–0.93	0.8	0.91	<0.001
Validation cohort	MLR	0.71	0.66–0.75	0.62	0.72	–
	Monocyte	0.62	0.57–0.66	0.72	0.55	0.041
	Lymphocyte	0.55	0.50–0.60	0.21	0.66	0.009
	Composite variable	0.89	0.86–0.92	0.88	0.76	<0.001

ROC, receiver operating characteristic; MLR, monocyte-to-lymphocyte ratio; AUC, area under curve; CI, confidence interval.

a Comparison of MLR with other indexes.

### Subgroup analysis

**Figure 6:**
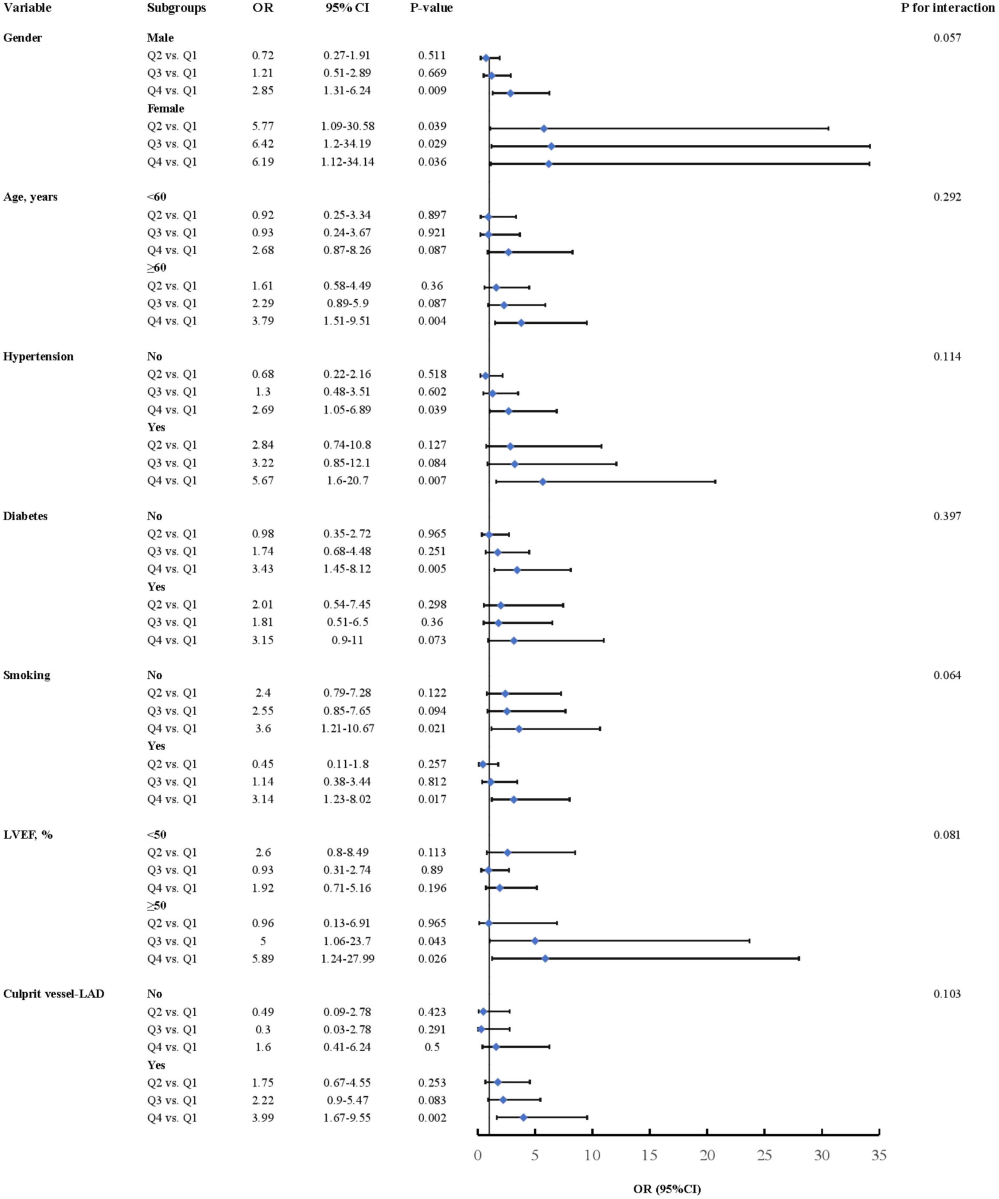
Subgroup analysis of the association between MLR and the risk for LVA formation in the first cohort. LVA, left ventricular aneurysm; LVEF, left ventricular ejection fraction; OR, odds ratio; CI, confidence interval.

**Figure 7:**
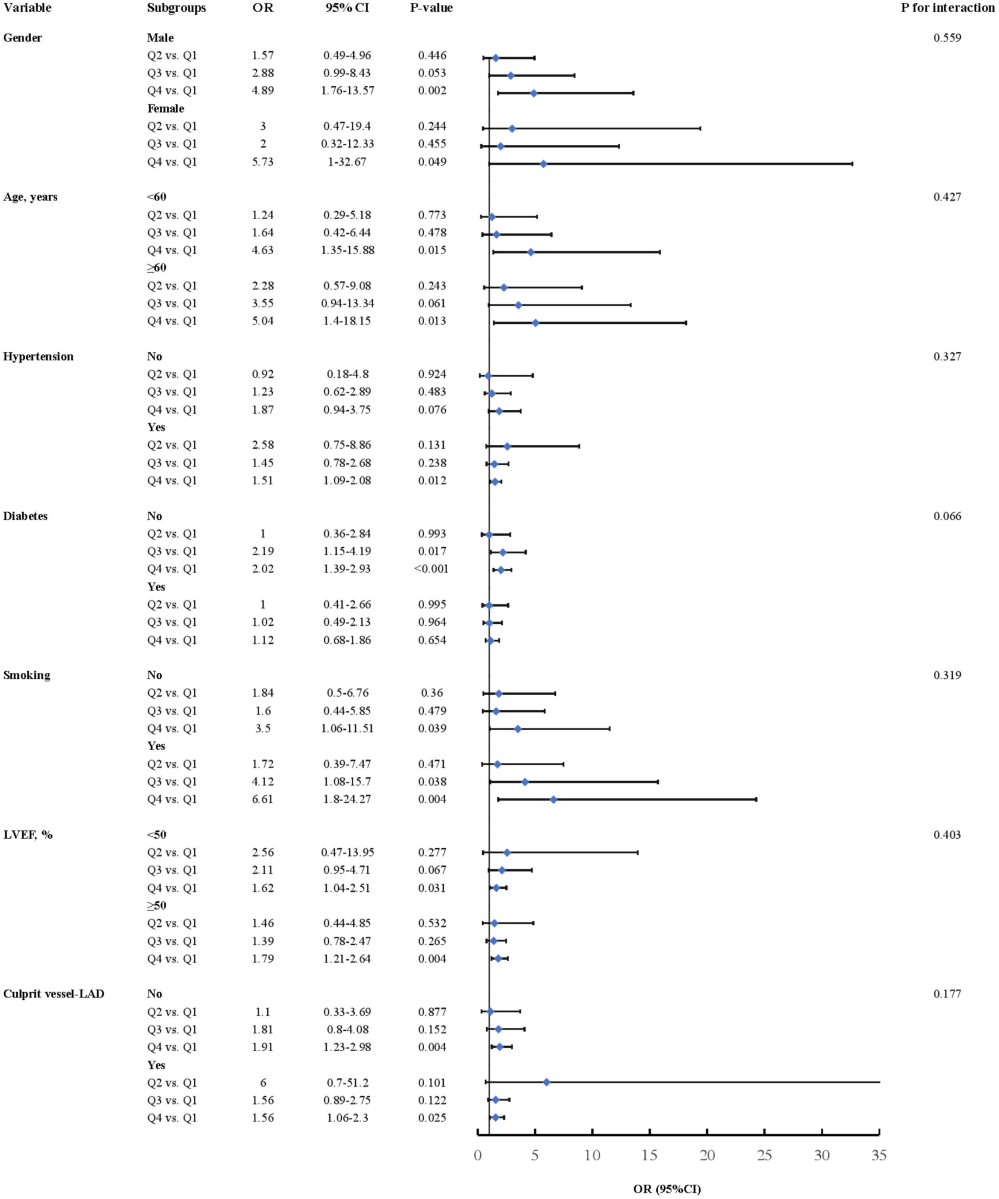
Subgroup analysis of the association between MLR and the risk for LVA formation in the validation cohort. LVA, left ventricular aneurysm; LVEF, left ventricular ejection fraction; OR, odds ratio; CI, confidence interval.

We conducted a subgroup analysis to further assess the independent predictive value of the MLR for LVA formation across various clinically relevant subgroups. In the first cohort, patients in the fourth quartile (Q4) exhibited an increased risk of LVA across all subgroups, except for those under 60 years of age, those with diabetes, those with LVEF <50%, and those without LAD as the culprit vessel, compared to patients in the first quartile (Q1) (**Figure 6**). In the validation cohort, comparable results were observed, with individuals in Q4 exhibiting an elevated risk of LVA across all subgroups, except for patients without hypertension and those with diabetes (**Figure 7**). Additionally, no significant interactive effects were observed between these clinically relevant variables and MLR on the risk of LVA (*P* for interaction >0.05).

## Discussion

In this research, we conduct the first comprehensive evaluation of the association between the monocyte-to-lymphocyte ratio (MLR) and the risk of LVA formation in patients with acute STEMI across two independent cohorts. Our findings demonstrate that an elevated MLR is associated with an increased risk of LVA formation. Additional analyses employing RCS confirmed the existence of a positive relationship between MLR and the likelihood of LVA, even after adjusting for various confounding factors. Furthermore, the predictive power of MLR for the risk of LVA outperformed that of both monocyte and lymphocyte. Remarkably, the composite variable indicated the most robust predictive strength.

Both monocytes and lymphocytes are integral components of the immune system, playing crucial roles in the regulation of immune responses and inflammation (20, 21). Nevertheless, existing research indicates that these cell types exhibit fundamentally opposing functions in the context of cardiovascular disease—for example, a cohort study by Yamamoto et al. identified a high monocyte count as an independent and incremental predictor of cardiovascular events in patients with coronary artery disease. Similarly, Han et al. studied 220 patients with aortic stenosis and found that an increased monocyte count was correlated with the rapid progression of the condition (22). Furthermore, Zhao et al., in a study of 8,943 patients with triple-vessel coronary artery disease, demonstrated that elevated monocyte levels were independent predictors of major adverse cardiovascular and cerebrovascular events (23). In contrast, a retrospective study by Carubelli et al. revealed that a low lymphocyte count in patients with acute heart failure was associated with heightened in-hospital mortality during the hospitalization period (12). Bian et al. demonstrated that a decreased lymphocyte percentage could function as an independent predictor for acute coronary syndrome upon admission and was linked to 1-year major adverse cardiac events during clinical follow-up in patients with coronary heart disease (13).

The divergent functions of monocytes and lymphocytes in cardiovascular events suggest that the MLR may offer superior predictive power for cardiovascular diseases. Previous research has established a strong association between MLR and cardiovascular events. A multicenter retrospective cohort study revealed that an elevated MLR was significantly linked to a hazard ratio (HR) of 1.45 for CVD mortality in patients undergoing peritoneal dialysis (24). In a study by Zhai et al. involving 5,512 patients in a cardiac intensive care unit, MLR was found to be independently associated with in-hospital mortality (25). Additionally, a positive correlation between MLR and the risk of myocardial infarction was observed in diabetic populations (26). In our current study, we similarly found that a higher MLR was significantly associated with an increased risk of LVA in patients experiencing acute STEMI. Furthermore, individuals in the highest MLR quartile (Q4) exhibited elevated levels of NT-proBNP, peak cTnI, and LDH, along with a reduced level of LVEF, compared to those in the lowest quartile (Q1) across both cohorts. These findings suggest that an elevated MLR is indicative of more severe coronary artery stenosis and myocardial injury. Moreover, the MLR exhibited superior predictive capability for LVA compared to monocytes and lymphocytes individually. Collectively, our findings highlight the critical role of MLR in cardiovascular diseases, corroborating previous research (14, 15, 27). Besides that, the DTB time was notably longer in the LVA group compared to the non-LVA group in our study, which was consistent with the result of a previous study that DTB time was associated with mortality and worse cardiac outcomes (28).

Numerous investigations have been carried out to pinpoint the risk factors associated with LVA—for instance, research by Peng et al. revealed that single-vessel disease, a reduced glomerular filtration rate, and abnormal ferritin levels serve as independent predictors of LVA development (4). However, the case–control approach utilized in their research constrained their capacity to establish causal connections. Conversely, our prospective study design bolsters the reliability and validity of our results. Zhang et al. discovered that the FIB-4 index acts as an independent predictor of LVA in STEMI patients undergoing PCI (29). Nevertheless, their study was conducted at a single center and lacked robust validation. Our study’s conclusions are derived from two independent cohorts, thereby providing more reliable and persuasive evidence. In a prospective cohort study involving 942 patients with acute anterior myocardial infarction treated with primary PCI, factors such as prolonged symptom-to-balloon time, elevated initial and residual SYNTAX scores, decreased LVEF, and ongoing ST segment elevation were recognized as independent predictors for LVA formation (7). While their research identified several risk factors for LVA, it was relatively broad in scope and did not concentrate on a specific risk factor for a more detailed and in-depth analysis, which somewhat undermines the credibility of the findings. In contrast, the current study delivers an extensive and detailed analysis of the relationship between MLR and LVA by accounting for confounding risk factors, examining restricted cubic spline (RCS) curves, and performing subgroup analyses, thereby yielding more robust and convincing outcomes.

The precise mechanisms underlying the association between the MLR index and LVA formation remain incompletely elucidated. Following myocardial infarction, circulating monocytes are recruited to the infarct zone, where they differentiate into macrophages (30). An elevated monocyte count may signify an exaggerated early pro-inflammatory response, characterized by the release of proteolytic enzymes, such as matrix metalloproteinases (MMPs), reactive oxygen species, and pro-inflammatory cytokines, including interleukin-1β (IL-1β) and tumor necrosis factor-alpha (TNF-α) (26). This persistent inflammatory environment can impair infarct healing, degrade the extracellular matrix, and weaken the myocardial scar, thereby creating a structural predisposition for diastolic and systolic bulging. Conversely, lymphocytes play a pivotal role in modulating inflammation and facilitating the transition to the reparative phase (31). Beyond local cardiac effects, the MLR may reflect a systemic pro-inflammatory state that influences remote organ systems and neurohormonal activation. This systemic inflammation can exacerbate endothelial dysfunction, promote hypercoagulability, and increase wall stress, all of which are factors that may secondarily exacerbate ventricular remodeling and elevate the risk of aneurysm formation.

Our study represents the inaugural investigation into the predictive value of the MLR for the risk of LVA in STEMI patients who have undergone primary PCI. In our analysis, we meticulously controlled for a range of confounding factors and examined the nonlinear relationship between MLR and LVA risk using RCS analysis, thus strengthening the credibility of our results. However, there are several limitations that should be acknowledged. Firstly, the identification of LVA relied on ultrasonographic assessments, which are not deemed the “gold standard” for detecting LVA. Nevertheless, this method is widely utilized in clinical settings and epidemiological research for LVA identification due to its accessibility and noninvasive characteristics. Secondly, even though the study had a considerable sample size, it focused solely on baseline levels of monocytes and lymphocytes, neglecting potential variations in MLR that might provide more profound understanding of the underlying mechanisms. Thirdly, potential selection bias may be present due to the retrospective nature of the study. To validate our results in future research, it is necessary to employ prospective study designs, utilize multimodal imaging validation, and implement core-laboratory adjudication. Fourth, our study was conducted during the COVID-19 pandemic, which may have affected hospital admissions, patient follow-up, and baseline inflammatory states. Although we excluded patients with active SARS-CoV-2 infection and both centers maintained protocol-driven STEMI care, unmeasured confounding related to pandemic-related changes in healthcare delivery cannot be entirely ruled out. Fifth, as an observational study, it cannot establish a causal relationship between MLR and LVA formation. While the temporal sequence supports a predictive role, we cannot definitively conclude whether MLR is a contributing cause, a consequence, or a biomarker sharing common upstream drivers with LVA. Sixth, in our subgroup analyses, the association between high MLR and increased LVA risk was not statistically significant in some subsets, such as patients under 60 years old, those with diabetes, or those without LAD as culprit artery. This may reflect limited statistical power within these smaller subgroups or suggest potential pathophysiological heterogeneity in the remodeling process across different patient profiles. While formal interaction tests were not significant, these findings highlight that the strength and consistency of the MLR–LVA association should be further validated in larger, phenotype-specific cohorts to fully define its generalizability. Finally, our analysis, while adjusting for several key clinical and angiographic variables, did not include all potential determinants of LVA such as precise ischemic time, detailed data on in-hospital arrhythmias, or genetic markers. The observed association of MLR with LVA should therefore be interpreted as one component within a complex pathogenic network.

## Conclusion

Our study presents evidence that an elevated MLR at admission is independently associated with subsequent LVA formation in STEMI patients treated with primary PCI. While its standalone discriminatory power is moderate, MLR represents a simple, early-available biomarker that may enhance the identification of high-risk patients. This finding raises the possibility of risk-stratified interventions, such as targeted anti-inflammatory therapy or enhanced monitoring for patients with elevated MLR. Future prospective studies and clinical trials are needed to determine whether modulating the inflammatory response reflected by MLR can effectively prevent this serious complication and improve long-term outcomes after STEMI.

## Data Availability

The original contributions presented in the study are included in the article/supplementary material. Further inquiries can be directed to the corresponding authors.
